# Exploring Quality Evaluation of Innovation and Entrepreneurship Education in Higher Institutions Using Deep Learning Approach and Fuzzy Fault Tree Analysis

**DOI:** 10.3389/fpsyg.2021.767310

**Published:** 2022-01-17

**Authors:** Changlin Wang, Puyang Zheng, Fengrui Zhang, Yufeng Qian, Yiyao Zhang, Yulin Zou

**Affiliations:** ^1^School of Economics and Management, Binzhou University, Binzhou, China; ^2^School of Educational Development, Nanchang University, Nanchang, China; ^3^College of Life Sciences, Sichuan Agricultural University, Yaan, China; ^4^School of Sciences, Hubei University of Technology, Wuhan, China; ^5^College of Art and Communication, Beijing Normal University, Beijing, China; ^6^The Third Clinical Medical College of China Three Gorges University, Gezhouba Central Hospital of Sinopharm, Yichang, China

**Keywords:** deep learning, fuzzy fault tree, teaching reliability, hyper-ellipsoid model, entrepreneurship classroom teaching

## Abstract

The quality of Innovation and Entrepreneurship Education (IEE) in higher institutions is closely related to the degree to which the undergraduates (UGs) absorb relevant innovation and entrepreneurship knowledge and their entrepreneurial motivation. Thus, an effective Evaluation of Educational Quality (EEQ) is essential. In particular, fault tree analysis (FTA), a common EEQ approach, has some disadvantages, such as fault data reliance and insufficient uncertainties handleability. Thereupon, this article first puts forward a theoretical model based on the deep learning (DL) method to analyze the factors of IEE quality; consequently, based on the traditional FTA, fuzzy fault tree analysis (FFTA) is proposed to evaluate the reliability of IEE classroom teaching for college teachers and students. Finally, based on the top event of entrepreneurial teaching failure, the hyper-ellipsoid model is implemented to restrict the interval probability of basic events and describe the deviation of uncertain events. Furthermore, the model accuracy is verified by a questionnaire survey (QS), based upon which the factors of IEE quality are analyzed. The results show that the designed QS has good reliability, validity, and fitness; the path coefficients of cooperative ability to critical thinking and innovative thinking are 0.9 and 0.66, respectively, indicating that the students’ cooperative ability plays a vital role in the classroom teaching. By calculation, the probability of “teaching failure” in entrepreneurial classroom teaching is 0.395, 3, 0.462, and 5. To sum up, the proposed method can effectively and quantitatively evaluate the quality of IEE in higher institutions, thus providing a certain basis for formulating relevant improvement strategies. The purpose is to provide important technical support for improving the IEE quality.

## Introduction

With the expanding recruitment of Chinese higher institutions, the undergraduate (UG) number increases year by year, and the pressure of job competition is increasing. In this case, entrepreneurship becomes one of the solutions to the problem for college graduates. Some data show that college graduates in China from about 5 million in 2010 to nearly 9 million in 2019, but the employment rate of college graduates is only about 70%, and less than 5% of college graduates choose to start their own businesses (Wei et al., 2019; Jena, 2020). Therefore, under the trend of “mass entrepreneurship and innovation,” perfecting the curriculum system related to entrepreneurship in higher institutions and improving the quality of UGs’ entrepreneurship become the focuses of scholars in relevant fields. Classroom teaching is a teaching method formed by integrating many related factors, such as educational objectives, teaching methods, teaching contents, and evaluation (Lee et al., 2018). The improvement of Innovation and Entrepreneurship Education (IEE) quality in higher institutions can promote UGs’ innovation and entrepreneurship ability and strengthen their entrepreneurial motivation. Therefore, it is necessary to evaluate the IEE quality in higher institutions to unveil the existing problems of the current teaching modes, based upon which targeted improvement strategies can be put forward to improve the employment rate of UGs (Li et al., 2020).

Literature review suggests that, at present, the checklist of the multi-index comprehensive evaluation method is often used to evaluate the reliability of IEE classrooms for college teachers and students. The advantage of this method is simplicity, easy operability, and satisfactory evaluation results (Adem et al., 2017; Chen, 2019). So far, scholars have evaluated the reliability of classroom teaching, mainly from the cultivation of learners’ learning interests and the stimulation of spontaneous learning awareness, which, however, belong to qualitative research and lack quantitative research. Today, the Evaluation of Educational Quality (EEQ) based on fault tree analysis (FTA) theory has become a hot research topic to improve the quality of classroom education and teaching in higher institutions. Relevant literature has focused on relatively simple methods, such as correlation analysis and descriptive statistics to evaluate the reliability of classroom teaching, which is a superficial analysis in terms of the factors of teaching quality and cannot understand its internal specific structure (Kermany et al., 2018; Rajkomar et al., 2018). The FTA method is proposed in the middle of the 20th century, which has a clear logic when evaluating the reliability of things, and intuitiveness, and high accuracy.

However, FTA relies heavily on relevant data to accurately determine the probability of the bottom event (Ravi et al., 2017; Yuge and Yanagi, 2017). In practical applications, fault data are often difficult to obtain; moreover, the system fault mechanism and the relationship between events are uncertain under fault tree with multiple bottom events; the failure probability (FP) ranges immensely after the top event analysis (Sang et al., 2019). Thereupon, this paper first puts forward a theoretical model based on the deep learning (DL) method to study the factors of IEE quality; then, based on the traditional FTA, fuzzy FTA (FFTA) is proposed to evaluate the reliability of IEE classroom teaching for college teachers and students. Finally, based on the top event of IEE failure, the hyper-ellipsoid model is used to restrict the interval probability of basic events and describe the deviation of uncertain events. Furthermore, the model accuracy is verified by a questionnaire survey (QS), and the factors of IEE quality are analyzed. The purpose is to provide important technical support for improving the IEE quality.

## Relevant Theoretical Analysis and Model Implementation

### Deep Learning

**FIGURE 1:**
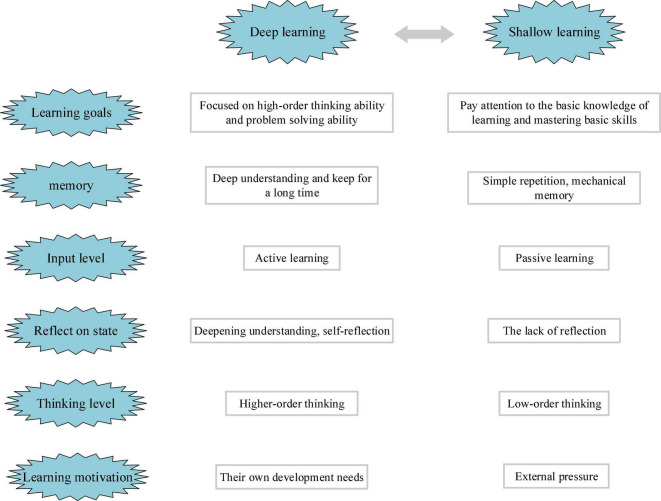
Comparison of deep learning (DL) and superficial learning.

Deep learning is a concept put forward by American scholars in the second half of the 20th century. Initially, DL aims to explore learners’ learning investment and mastery of knowledge. In the process of learning, learners will adopt different strategies to master knowledge. Learning methods can be simply divided into in-depth learning and superficial learning. Deep learners will think, understand, and put forward questions in the process of learning, whereas superficial learners do not pay attention to the understanding of knowledge but acquire knowledge through passive memory. Obviously, DL is superior to superficial learning (Vaurio, 2017). Furthermore, a comparison is made between in-depth learning and superficial learning, as shown in Figure 1 (Chen et al., 2017a,b).

**FIGURE 2:**
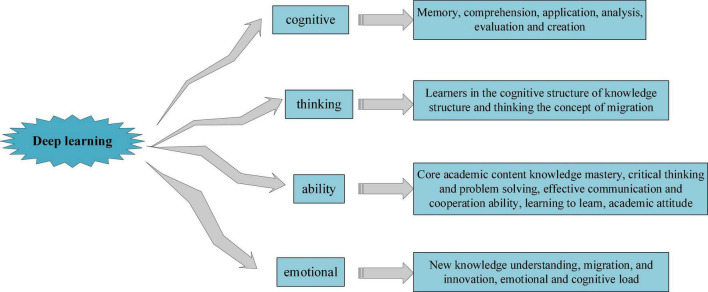
Definition of deep learning (DL) from different perspectives.

At present, there is no unified definition of DL, but based on relevant literature, scholars define DL mainly from the following four aspects, as shown in Figure 2 (Kim et al., 2017; Stillman et al., 2018; Chipamaunga and Prozesky, 2019; Ichsan et al., 2019).

### Implementation of Hypothesis Model in the Process of Deep Learning

**FIGURE 3:**
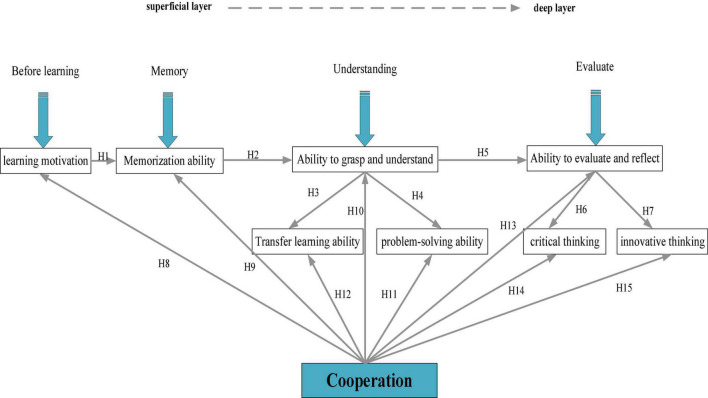
Hypothetical model of UGs’ DL process. DL, deep learning; UG, undergraduate.

Based on the relationship between factors of DL, a theoretical model is implemented, and the QS method is used to test the model in higher institutions to improve the teaching quality of IEE classrooms. The proposed DL hypothesis model is shown in Figure 3.

#### Learning Motivation

Learning motivation refers to the internal motivation of learners to carry out learning activities or maintain the existing learning state. The strength of learning motivation can be seen through learners’ learning initiative. The stronger learners’ learning initiative is, the stronger their learning motivation is. Moreover, learning motivation plays an important role in promoting learning. For example, in life and schools, when learned are rewarded every time they successfully remember some knowledge points, their learning process will be greatly promoted (Darwish et al., 2017; Yan and Jackson, 2017). Thereupon, the following hypothesis is put forward:

H1: Strong learning motivation improves learners’ memorization.

#### Superficial Learning

Superficial learning refers to the passive new knowledge learning to fulfill a task or avoid punishment, which mainly relies on memorization, comprehension, and understanding. Memorization, in particular, is the minimum requirement for learners in the cognitive process, which mainly refers to the ability to recall the mastered knowledge, methods, and theories. By contrast, comprehension and understanding are the in-depth perceptions of knowledge based on memorization, and the integration of old and new knowledge into applications (Liu et al., 2019; Yan et al., 2019). Hence, memorization can promote learners’ further mastery of knowledge. Accordingly, the following hypothesis is put forward:

H2: Strong memorization further promotes the perception of knowledge.

#### Deep Learning

When transferring knowledge, learners should have a deep understanding of knowledge (based on which hypothesis H3 is put forward). The ability to transfer knowledge is conducive to improving learners’ problem-solving ability (based on which hypothesis H4 is put forward). In Bloom’s cognitive structure, transferability and problem-solving ability, the ability to evaluate and solve problems, and critical thinking and innovative thinking belong to the stages of application analysis, evaluation, and synthesis (Wu and Song, 2019; Wu et al., 2020), and accordingly to which hypotheses H5, H6, and H7 are put forward, respectively.

H3: A deep understanding of knowledge is favorable to the transfer and application of knowledge.
H4: Strong knowledge transferability is favorable for learners to solve practical problems.
H5: Strong knowledge transferability is favorable to learners’ reflection on their learning process.
H6: The improvement of learners’ evaluation and reflection ability is favorable to the development of critical thinking.
H7: The improvement of learners’ evaluation and reflection ability is favorable to the development of innovative thinking.

In the process of learning, students can improve their abilities from all aspects through cooperative learning. For example, through cooperation, group members’ enthusiasm will be affected by each other, thereby improving their learning motivation (Hortiguela-Alcala et al., 2019; Namaziandost et al., 2019); based on this, hypothesis H8 is proposed. On the other hand, learners can also benefit from reviewing others’ views and memories, hence deepening their understanding and analyzing each other’s views; in this process, learners’ faculty of memory is enhanced (Amini, 2019), along with understanding (Sumarni et al., 2018), knowledge transference, and application (Indrayati, 2019), and problem-solving (Jalinus et al., 2019) skills; based on this, hypotheses H9, H10, H11, and H12 are put forward. Additionally, everyone has their unique ways of understanding and tackling problems, through the comparison of which, their ability to evaluate problems and reflect can be substantially boosted (Erdogan, 2019), and critical thinking (Huang et al., 2017; Sanchez-Hernandez et al., 2018) and innovative thinking (Hasan et al., 2019) capabilities; based on this, hypotheses H13, H14, and H15 are proposed. Therefore, the ability of cooperative learning is indispensable in the learning process. Thereupon, the following hypotheses are put forward:

H8: Cooperative learning improves learning motivation.
H9: Cooperative learning improves learners’ memorization.
H10: Cooperative learning improves learners’ ability to understand and comprehend knowledge.
H11: Cooperative learning improves learners’ knowledge transferability and application.
H12: Cooperative learning improves learners’ problem-solving ability.
H13: Cooperative learning improves learners’ evaluation and reflection ability.
H14: Cooperative learning improves learners’ critical thinking.
H15: Cooperative learning improves learners’ innovative thinking.

### Fuzzy Fault Tree Analysis

Under the traditional FTA, it is necessary to clarify the possibility of failure of each component, which is difficult under the current technologies. The commonly used logic gates in the traditional FTA to analyze the relationship between events are the AND gate and OR gate, but in practice, the relationship between events has great uncertainty (Deng et al., 2021; Liu and Chen, 2021). Therefore, the FFTA is introduced to make up for the shortcomings of the traditional FTA.

**FIGURE 4:**
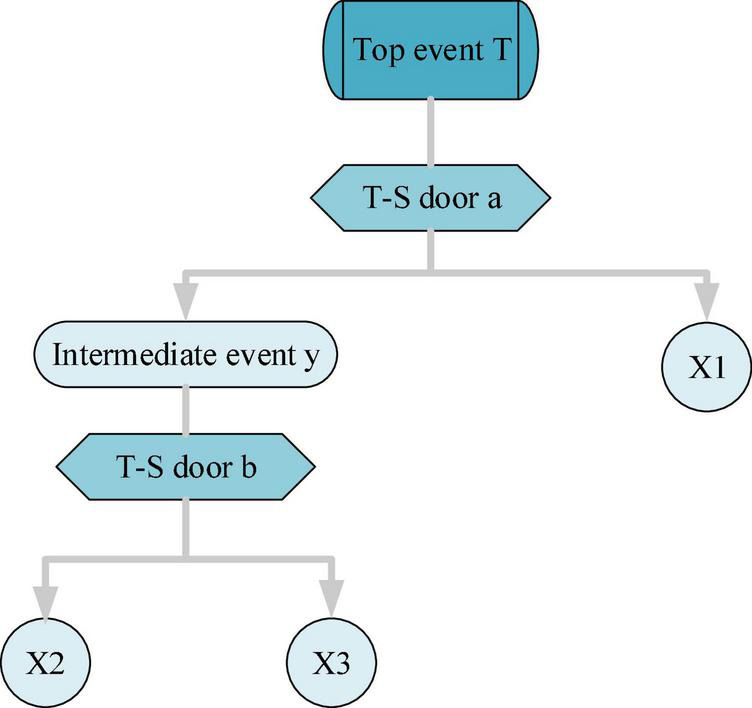
T–S fuzzy fault tree model.

Given the complexity and diversity of the relationship between actual events, a T–S fuzzy fault tree is constructed based on the traditional fuzzy number, which uses the T–S gate instead of the AND gate and OR gate. However, the specific algorithm for the importance of the T–S fuzzy fault tree is not yet available, and the importance of the algorithm for the traditional fault tree is extended in the relevant literature. Accordingly, the corresponding calculation of the T–S fuzzy fault tree is obtained (Wu et al., 2020; Liu et al., 2021). The model structure of the T–S fuzzy fault tree is shown in Figure 4:

In Figure 4, X1, X2, and X3 represent bottom events. The working principle of the fault tree model: the fault data of top events can be calculated through the T–S gate according to the upper fault data.

**FIGURE 5:**
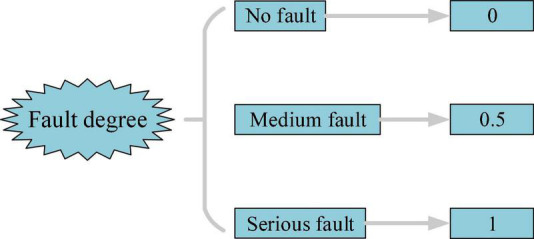
Fuzzy numbers and event description.

(1) Fuzzy number: When historical fault data are insufficient, or the system operating environment is unstable, the probability of component failure is uncertain. Therefore, this article adopts the concept of fuzzy numbers to describe these uncertain events, which can use the interval number in [0–1] to describe the fault degree of components, as shown in Figure 5.

(2) Model and algorithms: As a relatively fuzzy reasoning model, the T–S model uses few IF-THEN rules when constructing complex non-linear functions. Here, $\left(a_{1}^{1},a_{1}^{2},\ldots ,a_{n}^{k_{n}}\right)$, $\left(a_{n}^{1},a_{n}^{2},\ldots ,a_{n}^{k_{n}}\right)$, and (*b*^1^,*b*^2^,… *b^kn^*) present the fault degree of bottom event *a* and the top event *b*, respectively. Then, Equations (1) and (2) are obtained:

(1) $$\mu \,\left(F\right)=\left\{ \begin{array}{cc} 0, & 0\leq F\leq M_{0}-s_{l}-f_{l};\\ \frac{F-\left(M_{0}-s_{l}-f_{l}\right)}{f_{l}}, & M_{0}-s_{l}-f_{l}<F\leq M_{0}-s_{l}\\ 1, & M_{0}-s_{l}<F\leq M_{0}+s_{r}\\ \frac{\left(M_{0}+s_{r}-f_{r}\right)-F}{f_{r}}, & M_{0}+s_{l}<F\leq M_{0}+s_{r}+f_{r}\\ 0, & M_{0}+s_{r}+f_{r}<F\leq 0 \end{array}\right.$$

(2) $$\left\{ \begin{array}{c} 0\leq a_{1}^{1}<a_{1}^{2}<\cdots <a_{1}^{k_{1}}\leq 1\\ 0\leq a_{2}^{1}<a_{2}^{2}<\cdots <a_{2}^{k_{2}}\leq 1\\ 0\leq a_{n}^{1}<a_{n}^{2}<\cdots <a_{n}^{k_{n}}\leq 1\\ 0\leq b^{1}<b^{2}<\cdots <b^{k_{n}}\leq 1 \end{array}\right.$$

When rule *l*(*l* = 1,2,…,*n*) is given, if the fault degree of *a*_*1*_ is $a_{1}^{i_{1}}$, and $a_{2}=a_{2}^{i_{2}}$, then the probability of the fault of the top event with the degree of b1 and b2 is *P^l^*(*b*^1^) and *P^l^*(*b^k^n*), respectively, where *i*_1_ = 1,2,…,*k*_1_,…,*i*_*n*_ = 1,2,…,*k*_*n*_, the total number of *l* can be obtained, *m* = *k*_1_*k*_2_…*k*_*n*_.

The fuzzy possibility of failure of the bottom event is denoted as $P\left(a_{1}^{i_{1}}\right)\left(i_{1}=1,2,\ldots ,k_{1}\right),P\left(a_{1}^{i_{2}}\right)\left(i_{2}=1,2,\ldots ,k_{2}\right),\ldots ,P\left(a_{n}^{i_{n}}\right)\left(i_{n}=1,2,\ldots ,k_{n}\right)$. Then, the calculation of the fuzzy possibility of rule *l* reads:

(3) $$P_{0}^{l}=P\,\left(a_{1}^{i_{1}}\right)\,P\,\left(a_{1}^{i_{2}}\right)\,L\,P\,\left(a_{n}^{i_{n}}\right)$$

Based on this, the calculation of the fuzzy possibility of top events reads:

(4) $$\left\{ \begin{array}{c} P\,\left(b^{1}\right)=\sum_{i=1}^{m}P_{0}^{l}\,P^{l}\,\left(b^{1}\right)\\ P\,\left(b^{2}\right)=\sum_{i=1}^{m}P_{0}^{l}\,P^{l}\,\left(b^{2}\right)\\ \cdots \\ P\,\left(b^{n}\right)=\sum_{l=1}^{m}P_{0}^{l}\,P^{l}\,\left(b^{k_{n}}\right) \end{array}\right.$$

When the fault degree of the bottom event is known, the fuzzy possibility of the top event can be obtained according to the T–S model, as shown in Equation (5):

(5) $$\left\{ \begin{array}{c} P\,\left(b^{1}\right)=\sum_{l=1}^{m}\beta_{l}^{{}^{*}}\,\left(a^{\prime }\right)\,P^{l}\,\left(b^{1}\right)\\ P\,\left(b^{2}\right)=\sum_{l=1}^{m}\beta_{l}^{{}^{*}}\,\left(a^{\prime }\right)\,P^{l}\,\left(b^{2}\right)\\ \ldots \\ P\,\left(b^{n}\right)=\sum_{l=1}^{m}\beta_{l}^{{}^{*}}\,\left(a^{\prime }\right)\,P^{l}\,\left(b^{k_{n}}\right) \end{array}\right.$$

where

(6) $$\beta_{l}^{{}^{*}}\,\left(a^{\prime }\right)=\prod_{j=1}^{n}\mu_{a_{j}}^{i_{j}}\,\left(a_{j}^{\prime }\right)/\sum_{l=1}^{m}\prod_{j=1}^{n}\mu_{a_{j}}^{i_{j}}\,\left(a_{j}^{\prime }\right)$$

If the fuzzy possibility of bottom events is known, the fuzzy possibility of top events can be calculated according to the relevant rules of T–S and the fuzzy possibility of top events can be deduced according to the fault degree of bottom events.

### Hyper-Ellipsoid Model Theory

When describing the probability uncertainty of bottom events, the ellipsoid domain of the hyper-ellipsoid model can be used, and its size can describe the deviation degree of uncertain events. The set of ellipsoidal domains is composed of the probabilities of all underlying events in the fault tree, which can be described as in Equation (7):

(7) $$U:\sum_{i=1}^{n}\left(\frac{x_{i}-a_{i}}{b_{i}}\right)\leq 1$$

where a_*i*_ is the nominal value, and b_*i*_ is the deviation; x_*i*_ is the probability of occurrence of each bottom event. The calculations of a_*i*_ and b_*i*_ are shown in Equations (8) and (9):

(8) $$a_{i}=\frac{x_{i}^{\prime }+x_{i}^{{}^{\prime \prime }}}{2}$$

(9) $$b_{i}=\frac{x_{i}^{{}^{\prime \prime }}-x_{i}^{\prime }}{2}$$

The AND-gate interval operator and logic gate symbol based on the hyper-ellipsoid model are shown in Equations (10)–(13):

(10) $$x_{a\,n\,d}\in \left[x_{a\,n\,d}^{l},x_{a\,n\,d}^{u}\right]$$

(11) $$x_{a\,n\,d}^{l}=\min\,\prod_{i=1}^{n}x_{i}$$

(12) $$x_{a\,n\,d}^{u}=\max\,\prod_{i=1}^{n}x_{i}$$

which satisfies Equation (13):

(13) $$\sum_{i=1}^{n}{\left(\frac{x_{i}-a_{i}}{b_{i}}\right)}^{2}\leq 1$$

The interval operator and logic gate symbol of the OR gate is shown in Equations (14)–(17):

(14) $$x_{o\,r}\in \left[x_{o\,r}^{l},x_{o\,r}^{u}\right]$$

(15) $$x_{o\,r}^{l}=\min1-\prod_{i=1}^{n}\left(1-x_{i}\right)$$

(16) $$x_{o\,r}^{u}=\max1-\prod_{i=1}^{n}\left(1-x_{i}\right)$$

which satisfies Equation (17):

(17) $$\sum_{i=1}^{n}{\left(\frac{x_{i}-a_{i}}{b_{i}}\right)}^{2}\leq 1$$

### Questionnaire Survey Design and Implementation Method

#### Research Subject

This section surveys teachers and students from Yunnan S University, a comprehensive university with both Liberal Arts and Science and Engineering majors. Specifically, the Literature, History, Philosophy, Economics, Management, Law, Education, and Art are classified as Liberal Arts majors, whereas Science, Engineering, Agriculture, and Medicine are classified as Science and Engineering majors. Overall, 300 Liberal Arts and Science and Engineering students, covering freshmen to seniors, are selected together with 20 teachers.

#### Preparation of Questionnaire Survey

**FIGURE 6:**
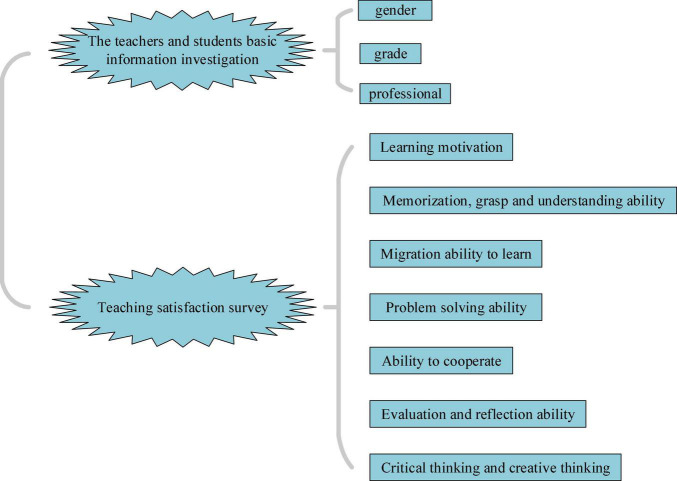
QS design of IEE classroom teaching process for college teachers and students. QS, questionnaire survey; IEE, Innovation and Entrepreneurship Education.

The purpose is to evaluate the IEE quality in higher institutions. Under the background of DL, the *QS on the IEE classroom teaching process of college teachers and students* is devised based on the QS of DL subscale. The QS includes two parts, as shown in Figure 6.

Figure 6 displays that the QS consists of two parts: basic information of teachers and students and teaching satisfaction, totaling 42 questions: 3 and 39 in the first and second parts, respectively, and the second part involves nine dimensions. Likert’s 5-point scale is employed for the QS scoring, and the numbers 1–5 are used to represent “completely inconsistent” to “fully consistent,” respectively (Liu et al., 2021). The specific QS is illustrated in the Supplementary Appendix.

#### Formal Distribution and Recovery of Questionnaire Survey

Totally, 320 QSs are distributed, 310 ones are recovered, and the eight invalids are excluded, including 302 valid QSs, with an effective recovery rate of 94.38%. Then, IBM SPSS 22.0 (IBM SPSS, Armonk, NY, United States) is used to process and analyze the data. The main statistical methods include descriptive analysis, independent sample *t*-test, ANOVA, correlation analysis, and regression analysis. The basic feature distribution of samples is presented in Table 1.

**TABLE 1:** Basic features of QS samples.

	Gender		Grade				Major	
	Male	Female	Freshmen	Sophomore	Junior	Senior	Liberal Arts	Science and Engineering
Population	72	230	78	82	65	77	159	143
Proportion	23.84%	76.16%	25.83%	27.15%	21.52%	25.50%	52.65%	47.35%

#### Reliability and Validity

Reliability and validity test of the QS: SPSS is employed for reliability analysis and factor analysis of the QS. After calculation, the Kaiser Meyer Olkin (KMO) value is 0.973, close to 1, and sig (0.000) is significant.

Cronbach’s α coefficient is used to test the reliability of the nine dimensions in the second part of the QS. The specific calculation reads as follows:

(18) $$A=\frac{k}{k-1}\,\left(1-\frac{\sum_{i=1}^{k}S_{i}^{2}}{S_{x}^{2}}\right)$$

where K is the number of topics; $S_{x}^{2}$ is the variance of the total score; $S_{i}^{2}$ is the variance of the score of question i.

**FIGURE 7:**
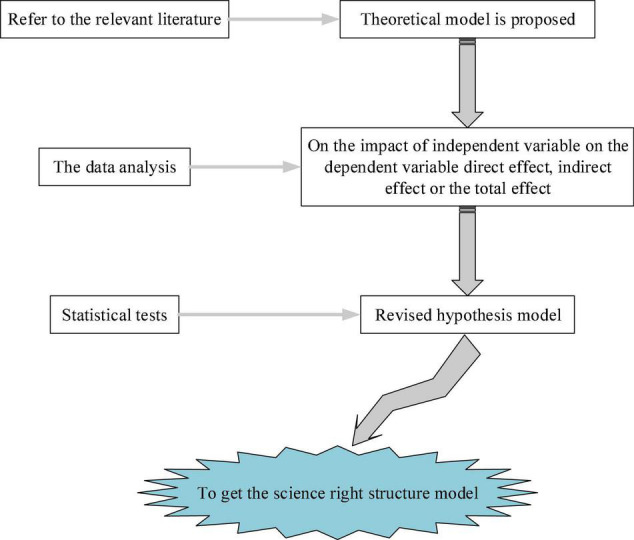
The basic process of structural equation model statistics.

Furthermore, IBM SPSS 25.0 and AMOS 21.0 software (IBM SPSS, Armonk, NY, United States) are used to analyze the collected QS data, and the structural equation model is used to test the hypothetical model. The structural equation model integrates the two statistical methods of path analysis and factor analysis. The main process is shown in Figure 7.

Chi-square degree of freedom (DOF) ratio, square root of the mean square sum of progressive residuals (RESEA), fitting index (FI), and comparative fitting index (CFI) are commonly used in structural equation models, which are specifically selected here to test the fitting degree of the model used in this study.

Chi-square DOF ratio (χ^2^/*df*): Chi-square χ^2^ indicates the fitting degree between the variables in the constructed model and the obtained data. When χ^2^ = 0, the fitting effect is the best, and then, the matching degree between the variables in the model and the observed data is the highest. The specific calculation equations read:

(19) $$\chi^{2}=\left(n-1\right)\,F\,\left(S;\Sigma \right)$$

(20) $$F\,\left(S;\Sigma \right)=t\,r\,\left(S\,\Sigma^{\left(-1\right)}\right)+\mathrm{lg}\left|\Sigma \right|-\mathrm{lg}\left|S\right|-P$$

where S is the matrix sequence constructed by the obtained data; Σ is the matrix constructed by the hypothesis model; Σ(0) is the sum of the elements on the diagonal in the matrix. The number of variables and data samples will affect the value of χ. Therefore, this paper uses χ^2^/*df* to judge the fitting degree of the model.

RESEA: the specific calculation of RESEA reads:

(21) $$R\,E\,S\,E\,A=\sqrt{\frac{F_{0}}{d\,f}}$$

where F_0_ is the value of the difference function; df is the degree of freedom. The smaller RESEA is, the better the fitting effect between the representative data is.

Fitting indexes (GFI/AGFI): GFI refers to goodness-of-fit index; adjusted GFI (AGFI) adjusts the fitting index to eliminate the influence of DOF in GFI. The calculation of GFI and AGFI reads as follows:

(22) $$G\,F\,I=1-\frac{F\,\left(S;\Sigma \right)}{F\,\left(S;\Sigma \,\left(0\right)\right)}$$

(23) $$A\,G\,F\,I=1-\left(1-G\,F\,I\right)\,\left[\frac{n\,\left(n+1\right)}{2\,d\,f}\right]$$

where S is the matrix sequence constructed with actual data; Σ is the matrix sequence constructed according to the hypothesis model; Σ(0) is the independent matrix; df is the degree of freedom. Generally, when GFI and AGFI are greater than 0.9, the model has a good fitting effect.

CFI: the calculation of CFI reads:

(24) $$C\,F\,I=1-\frac{M\,A\,X\,\left(\chi_{T}^{2}-d\,f_{T,0}\right)}{M\,A\,X\,\left(\chi_{N}^{2}-d\,f_{N,0}\right)}$$

where $\chi_{T}^{2}\,\chi_{N}^{2}$–χ^2^ between the hypothesis model and the actual data; df_*T*_ and df_*N*_ are the degrees of freedom between the hypothesis model and actual data. Generally, when CFI > 0.9, the fitting effect of the data is good, and the fitting effect is the worst at CFI = 0.8.

## Reliability Evaluation and Analysis of Innovation and Entrepreneurship Education Classroom Teaching

### Questionnaire Survey Result and Hypothesis Model Test

#### Statistical Results

**FIGURE 8:**
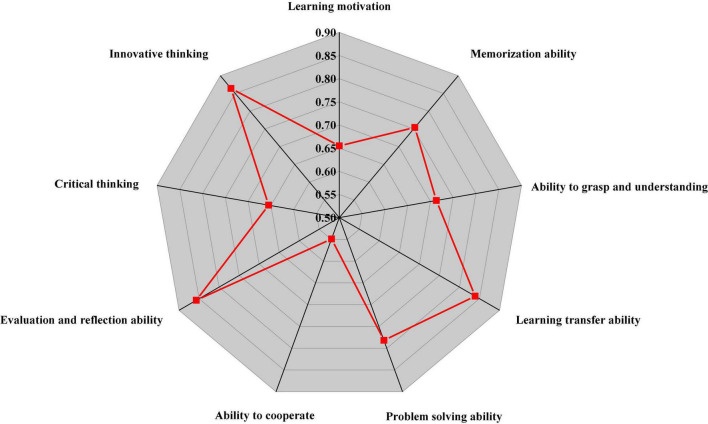
Cronbach’s α coefficient of each dimension in the questionnaire survey (QS).

IBM SPSS 25.0 (IBM Corp., Armonk, NY, United States) is used to analyze the nine dimensions in the QS, and the Cronbach’s α coefficient is obtained, as shown in Figure 8.

Factor analysis of the QS shows that the validity of the QS is 75.18%. Thus, the QS on the IEE classroom teaching of college teachers and students has good reliability and validity and can effectively evaluate the reliability of IEE classroom teaching.

#### Measurement Model Fitting Test Results

**FIGURE 9:**
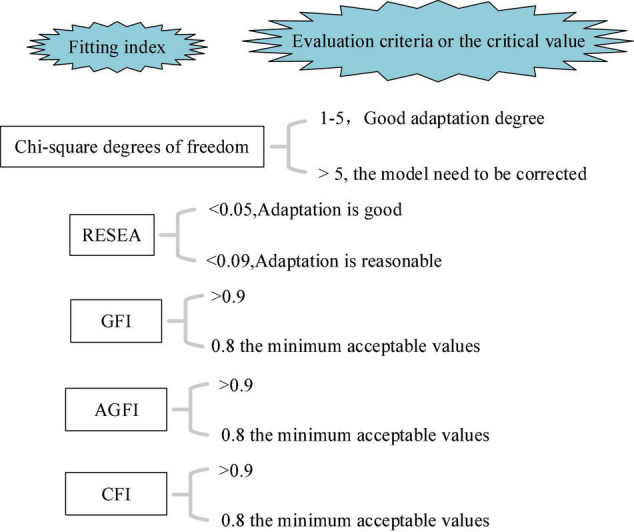
Evaluation criteria of model fitting degree.

**FIGURE 10:**
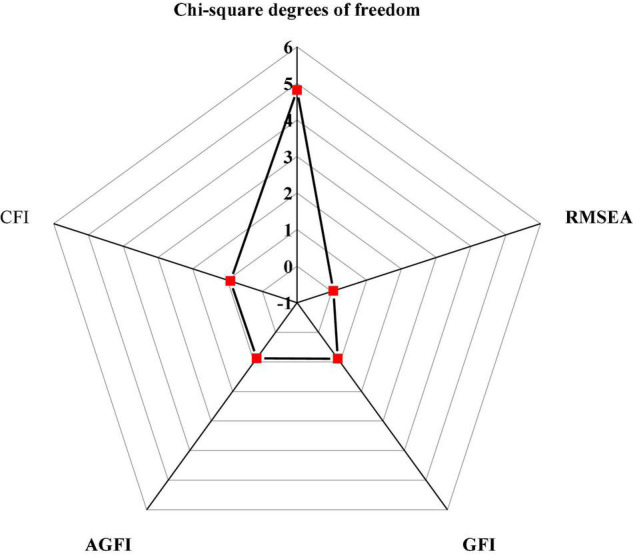
Hypothesis model fitting value.

The hypothesis testing mainly tests the chi-square DOF ratio, RESEA, GFI, and CFI of the hypothetical model. AMOS 21.0 is used to analyze 302 QSs collected. The evaluation criteria and fitting results of the model fitting degree are shown in Figures 9, 10 (Abebe, 2019; Atitsogbe et al., 2019; Abulela and Davenport, 2020; Liu, 2021).

RESEA,-square root of the mean square sum of progressive residuals; GFI, the goodness of fit index; AGFI, adjusted GFI; CFI, comparative fitting index.

Figure 10 displays that the value of χ^2^/*df* is 4.819, which is between 1 and 5, indicating a good degree of fitness; the value of RMSEA is 0.046, which is less than the critical value of 0.05; the values of CFI, AGFI, and CFI are 0.896, 0.882, and 0.917, respectively, which are greater than 0.8. The above data show that the theoretical model based on the DL process has a good fitting effect with the data from the actual investigation.

#### Structural Model Test

**FIGURE 11:**
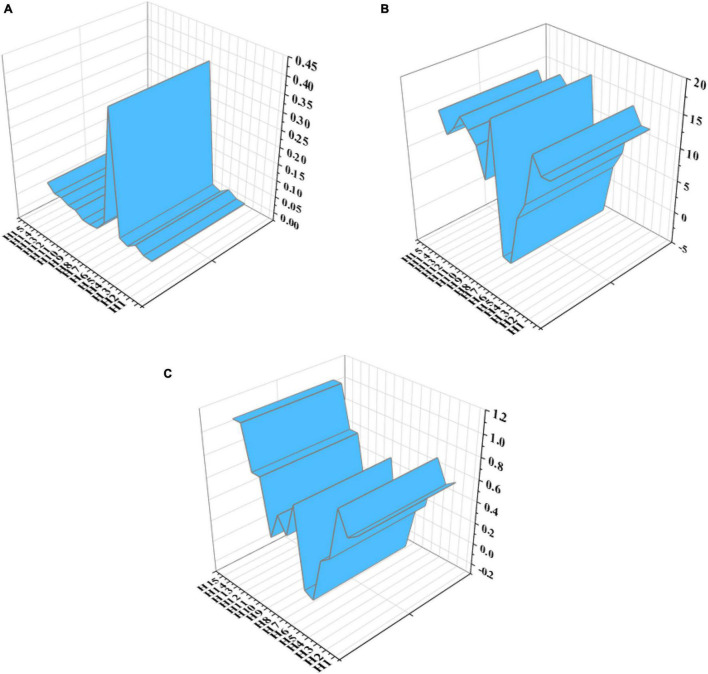
Parameter test value of the structural model. **(A)** Test value of S.E. **(B)** Test value of C.R. **(C)** Test value of β).

According to the fitting results of the hypothesis model in Figure 10, the path in the hypothesis model is tested, and the parameter significance and rationality test are taken as the test criteria. The results are shown in Figure 11.

Figure 11 illustrates that all 15 hypotheses paths meet the significance test, but the C.R. values of paths H6 and H7 are − 2.514 and − 2.244, respectively, and the β values are − 0.18 and − 0.14, respectively, which are negative, indicating that the influence of independent variables on dependent variables is negative; That is, “The enhancement of learners’ evaluation and reflection ability will not promote the development of critical thinking and innovative Thinking,” which is contrary to the original hypothesis, so the hypotheses paths H6 and H7 are not tenable.

**FIGURE 12:**
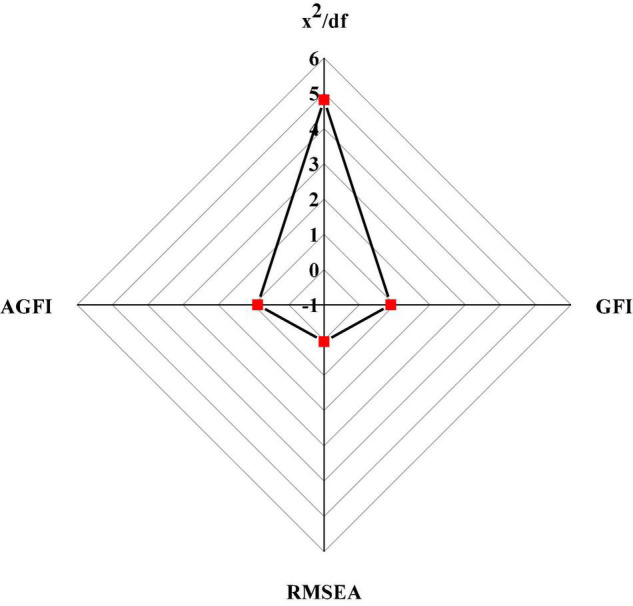
Fitting value of the modified structural model.

Since the hypotheses paths H6 and H7 are not tenable, these two paths are deleted, and the hypothetical model is revaluated. The fitting value of the modified structural model is shown in Figure 12.

**FIGURE 13:**
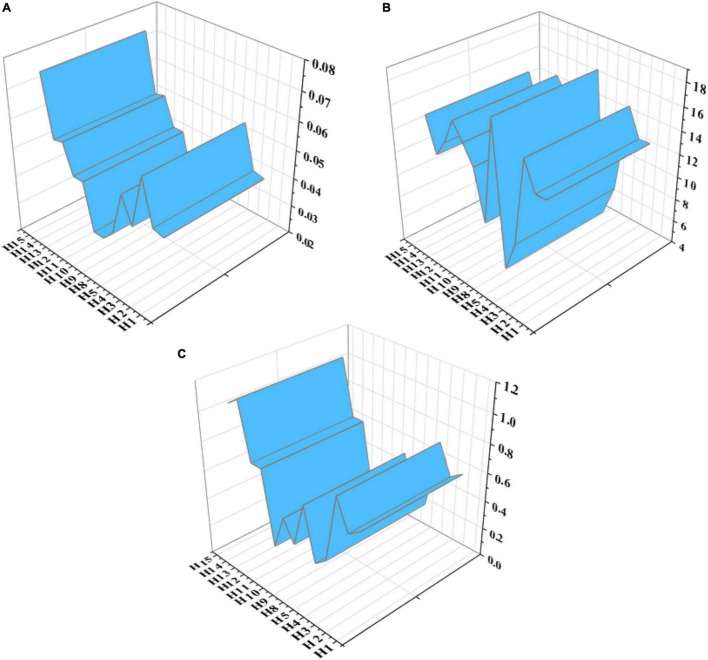
Parameter test value of the modified structural model. **(A)** Test value of S.E. **(B)** Test value of C.R. **(C)** Test value of β).

The parameter values of each path of the modified structural model are shown in Figure 13.

Figure 13 shows that the significance test and rationality test of each path of the modified structural model meet the critical value, and the test result is valid.

#### Research Results

**FIGURE 14:**
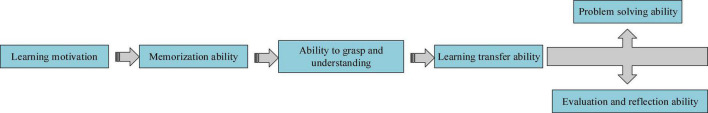
Two paths for UGs’ DL. DL, deep learning; UG, undergraduate.

In summary, there is a specific correlation between the nine factors of the DL process. Through analysis, the following two ways can be concluded to promote UGs’ DL and improve the efficiency of IEE classroom teaching in higher institutions, as shown in Figure 14:

Figure 14 reveals that in the process of IEE classroom teaching in higher institutions, learners’ problem-solving ability and evaluation and reflection ability will be significantly affected by learning motivation, but the above two learning paths have not developed learners’ critical and innovative thinking. Learning motivation plays a crucial role in learners’ learning process and is a prerequisite for superficial learning and DL. Before learners reach DL, they must go through superficial learning. After calculation, the path coefficients of cooperative ability to critical and innovative thinking are 0.9 and 0.66, respectively, indicating that learners’ cooperative ability promotes critical and innovative thinking.

To sum up, in the IEE classroom teaching, UGs generally lack critical and innovative thinking. At the same time, learners’ learning motivation and cooperation ability are the keys promoting the development of learners’ critical and innovative thinking.

### Reliability Analysis of Innovation and Entrepreneurship Education Classroom for College Teachers and Students Based on Fuzzy Fault Tree

#### Construction of Fuzzy Fault Tree of Innovation and Entrepreneurship Education Classroom System

**FIGURE 15:**
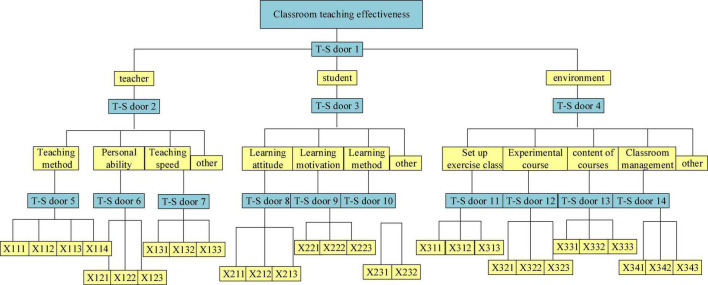
Fuzzy fault tree of classroom teaching effect.

**FIGURE 16:**
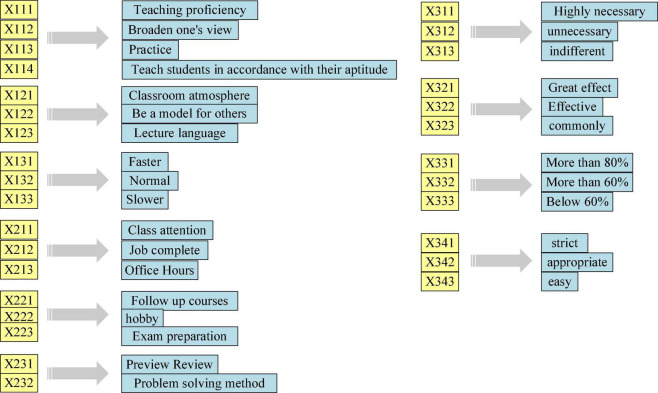
Fuzzy fault tree bottom events.

Furthermore, relevant literature is reviewed, based on which the fault tree model structure is constructed, as shown in Figures 15, 16.

Figures 15, 16 show that the top event is “classroom teaching effectiveness.” The causes of classroom teaching failure can be analyzed from three points: teachers, students, and environment, which are regarded as the intermediate events of the fault tree (Zheng and Ke, 2020; Zheng and Liu, 2020). Then, the factors affecting teachers, students, and environment are found to determine the bottom event of the fault tree, including ten aspects: students’ learning attitude, motivation, and methods; teachers’ teaching level, teaching methods, and personal charm, and also teaching content; the establishment of experimental class, exercise class, and classroom management (Zheng et al., 2015).

**FIGURE 17:**
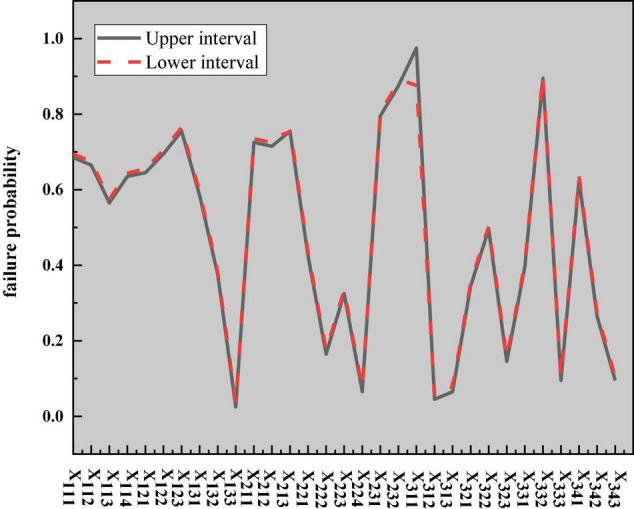
Probability of each bottom event of IEE classroom teaching effectiveness in higher institutions. IEE, Innovation and Entrepreneurship Education.

The logical relationship between events can be described by logic gates. Since the completion of the top event requires the joint action of intermediate events, such as teachers, students, and the environment, any problem in any intermediate link may affect the normal progress of the top event, and the lower-layer events also jointly affect their upper-layer events. AND-gate structure is used to connect the relationship between intermediate events and bottom events. After the basic events are simplified, the lower-layer bottom events are obtained. The bottom events are composed of questions that students can easily answer. Because these questions are mutually exclusive, the OR-gate structure is selected to connect the lower-level bottom events. The probability interval of the bottom events is determined according to the above-established model and ellipsoid model theory, as shown in Figure 17.

#### Probability Analysis of Classroom Teaching Effectiveness of Entrepreneurship in Higher Institutions Based on Ellipsoid Model

**FIGURE 18:**
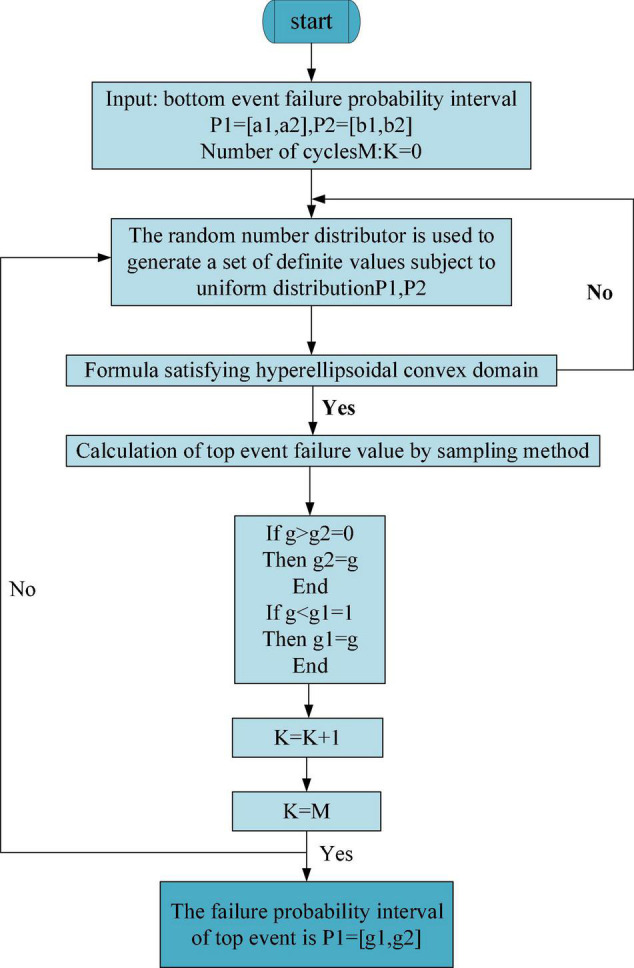
Monte Carlo sampling flowchart under the hyper-ellipsoid model.

Here, the double-layer Monte Carlo sampling simulation method is selected to calculate the probability of “classroom teaching failure” for the top events. The Monte Carlo sampling simulation estimates and describes the statistics of the function after sampling or simulation test of random variables and then obtains the approximate solution of engineering technical problems (Yuan and Wu, 2020; Xue and Deng, 2021). The main difference between the hyper-ellipsoid model and the interval model lies in outer sampling. When an interval model is used to sample the FP interval for bottom events, samples do not need to be screened, and they are regarded as effective samples. Under the hyper-ellipsoid model, samples are evaluated through the hyper-ellipsoid convex region equation. If the conditions are not met, it is necessary to resample. The FP estimation process based on the hyper-ellipsoid model is shown in Figure 18.

**FIGURE 19:**
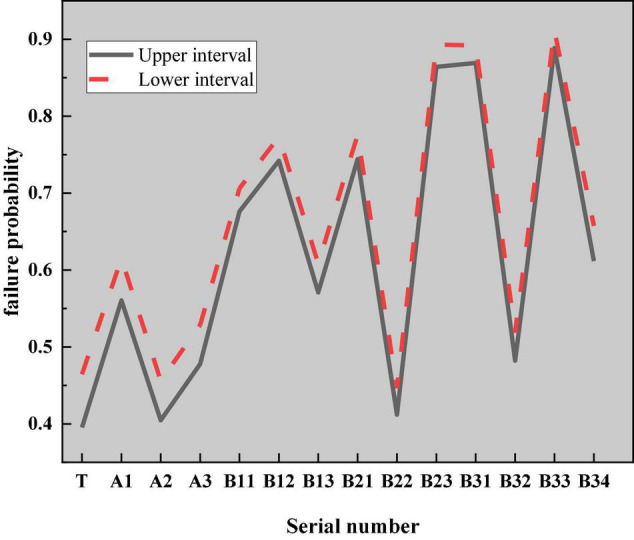
Event probability diagram of each layer.

According to the Monte Carlo sampling flowchart under the hyper-ellipsoid model, the reliability of IEE classrooms for college teachers and students is analyzed. According to the structure of the constructed fuzzy fault tree, after the probability of occurrence of the bottom event is determined, the probability of occurrence of the corresponding top event can be calculated according to the fault tree under the hyper-ellipsoid model. The specific calculation results are shown in Figure 19.

## Discussion

As a carrier of implementing IEE, the IEE curriculum has been given much concern. The main content of IEE only involves the connotation and significance of entrepreneurship, entrepreneurship spirit, and entrepreneurship laws and policies. Through the organic integration of innovative and entrepreneurial courses and professional curriculum systems, innovative and entrepreneurial practice is effectively linked to teaching practice (Almahry et al., 2018; Fiore et al., 2019). A theoretical model is designed by using DL, and an ellipsoid model is established based on FFTA. Through reliability analysis, it is found that UGs generally lack critical and innovative thinking, and students’ motivation for study and their cooperation ability are the key to promote the development of their critical and innovative thinking. Therefore, teachers should pay attention to the cultivation of students’ critical thinking and the enhancement of student’s learning motivation and cooperation ability, and appeal to parents and relevant social departments to join in the entrepreneurial education and provide a real entrepreneurial environment for students.

It is necessary to establish a diversified teaching system that combines IEE with professional education, and student’s ability to innovate, discover entrepreneurial opportunities, and carry out entrepreneurial practice should be improved. The basic courses of IEE should be wider, more targeted, and practical (Boldureanu et al., 2020). Through investigation and analysis, the theoretical model designed can be applied to practice, and the ellipsoid model can be used to analyze the reliability of entrepreneurial classroom teaching. The results show that the path coefficients of cooperative ability to critical thinking and innovative thinking are 0.9 and 0.66, respectively, indicating that students’ cooperative ability plays a vital role in classroom teaching. After calculation, the probability of “teaching failure” in entrepreneurial classroom teaching is (0.3953, 0.4625). Therefore, the use of DL and FFTA can accurately evaluate the reliability of IEE classroom teaching in higher institutions, which is helpful to improve the quality of IEE classroom teaching.

## Conclusion

To improve the quality of IEE classroom teaching for college teachers and students, this study uses the QS method and software engineering method to evaluate the reliability of IEE classrooms for college teachers and students based on DL and FFTA, respectively; then, the model accuracy is verified by QS, and the factors of IEE quality are analyzed. The results show that: (1) the designed QS has good reliability, validity, and fitness; (2) the evaluation outcome suggests that the path coefficients of cooperative ability to critical thinking and innovative thinking are 0.9 and 0.66, respectively, indicating that students’ cooperative ability plays a vital role in classroom teaching; and (3) after calculation, the probability of “teaching failure” in the IEE classroom is (0.3953, 0.4625). Through comprehensive analysis, the conclusions are summarized below:

(1) After the theoretical model of DL is established and tested, factors from nine dimensions affect the quality of IEE classroom teaching, and there is a specific correlation between these nine factors in structure and quantity. In classroom teaching, learners’ learning motivation and cooperative ability play a crucial role in their DL.
(2) The teaching process is analyzed based on the fuzzy fault tree and calculated by the Monte Carlo simulation method. The probability interval of the top event of IEE classroom teaching failure of college teachers and students is (0.3953, 0.4625), thereby quantitatively evaluating classroom teaching reliability.

Some shortcomings might need further exploration and adjustment: (1) only teachers and students of S University in Yunnan Province are selected for experimental research, so under the limited data scale, the research results lack representative; and (2) the model variables in FFTA are not explicitly analyzed. In the follow-up, it is expected to expand the sample size to verify and improve the theoretical model to obtain more objective and accurate evaluation; meanwhile, the appropriate fuzzy variables will be selected along with membership functions according to the actual situation, which is of great significance to improve the reliability of IEE classroom teaching. The purpose is to provide important technical support for improving the IEE quality.

## Data Availability Statement

The raw data supporting the conclusions of this article will be made available by the authors, without undue reservation.

## Ethics Statement

The studies involving human participants were reviewed and approved by Binzhou University and Gezhouba Central Hospital of Sinopharm Ethics Committee. The patients/participants provided their written informed consent to participate in this study. Written informed consent was obtained from the individual(s) for the publication of any potentially identifiable images or data included in this article.

## Author Contributions

All authors listed have made a substantial, direct, and intellectual contribution to the work, and approved it for publication.

## Conflict of Interest

The authors declare that the research was conducted in the absence of any commercial or financial relationships that could be construed as a potential conflict of interest.

## Publisher’s Note

All claims expressed in this article are solely those of the authors and do not necessarily represent those of their affiliated organizations, or those of the publisher, the editors and the reviewers. Any product that may be evaluated in this article, or claim that may be made by its manufacturer, is not guaranteed or endorsed by the publisher.
