# Supplementary material for: Exploring Quality Evaluation of Innovation and Entrepreneurship Education in Higher Institutions Using Deep Learning Approach and Fuzzy Fault Tree Analysis
Source: Front Psychol. 2022 Jan 17;12:767310. doi: 10.3389/fpsyg.2021.767310 (PMC8802833; doi:10.3389/fpsyg.2021.767310)
Supplement: Supplementary file 1 [file Data_Sheet_1.docx]

**Appendix**

QS on EEQ of IEE in higher institutions

Hello! First of all, thank you for your support for the QS. The purpose of this QS is to evaluate the quality of IEE in higher institutions. The QS is conducted anonymously. There is no right or wrong answer. The answer you choose will not have any adverse impact on you. You are expected to carefully fill in the questions in the QS. Your cooperation will be of great help to the research on the IEE quality evaluation in higher institutions. Thank you for your sincere cooperation!

1. Basic information: please mark "√" on the corresponding answer according to your own situation.

| Basic information | | | | |
| --- | --- | --- | --- | --- |
| 1. Gender. | Male □ | | Female □ | |
| 2. Grade. | Frenchmen □ | Sophomore □ | Junior □ | Senior □ |
| 3. Major. | Liberal Arts □ | | Science and Engineering □ | |

2. QS: please read the following questions carefully, then choose one answer that is closer to your actual situation from the five alternatives, and mark "√" on the corresponding one.

| Topic content | Completely inconsistent | Not so consistent | Not sure | Relatively consistent | Completely consistent |
| --- | --- | --- | --- | --- | --- |
|  | 1 | 2 | 3 | 4 | 5 |
| 1. Do you think you often associate, analyze, and compare the newly learned knowledge and concepts with the learned ones? | □ | □ | □ | □ | □ |
| 2. Do you think you will use knowledge to explain some everyday phenomena or solve some practical problems? | □ | □ | □ | □ | □ |
| 3. Do you think you will formulate diverse problem-solving strategies simultaneously and make reasonable decisions? | □ | □ | □ | □ | □ |
| 4. Do you think you try out various methods and ideas against problems? | □ | □ | □ | □ | □ |
| 5. Do you think you adopt a reverse perspective given a problem? | □ | □ | □ | □ | □ |
| 6. Do you think you always find another way to do things? | □ | □ | □ | □ | □ |
| 7. Do you think you can come up with new and different ideas? | □ | □ | □ | □ | □ |
| 8. Do you think you are an independent thinker? | □ | □ | □ | □ | □ |
| 9. Do you think you have a better imagination than others? | □ | □ | □ | □ | □ |
| 10. Do you think you cannot help asking questions throughout the learning process? | □ | □ | □ | □ | □ |
| 11. Do you think you like to solve problems with novel solutions? | □ | □ | □ | □ | □ |
| 12. Do you think the questions you raised are worth exploring? | □ | □ | □ | □ | □ |
| 13. Do you think you actively participate in group activities? | □ | □ | □ | □ | □ |
| 14. Do you think you will make preparations in advance and bring all the required materials for cooperation tasks? | □ | □ | □ | □ | □ |
| 15. Do you think you help the team solve any problems they face? | □ | □ | □ | □ | □ |
| 16. Do you think you help the team unite? | □ | □ | □ | □ | □ |
| 17. Do you think you share your ideas with the group? | □ | □ | □ | □ | □ |
| 18. Do you think can finish the team task on time? | □ | □ | □ | □ | □ |
| 19. Do you think you do not need to be reminded to do team tasks? | □ | □ | □ | □ | □ |
| 20. Do you think you learn from the rest of the team? | □ | □ | □ | □ | □ |
| 21. Do you think you will praise a teammate who has done well? | □ | □ | □ | □ | □ |
| 22. Do you think you listen carefully to your teammates? | □ | □ | □ | □ | □ |
| 23. Do you think you carefully consider everyone's ideas? | □ | □ | □ | □ | □ |
| 24. Do you think you always preview before class? | □ | □ | □ | □ | □ |
| 25. Do you think you will ask the teacher when you have a question in class? | □ | □ | □ | □ | □ |
| 26. Do you think you have learned from your homework mistakes? | □ | □ | □ | □ | □ |
| 27. Do you think you prefer challenging classes so that you can learn new things? | □ | □ | □ | □ | □ |
| 28. Do you think you set long-term goals for yourself? | □ | □ | □ | □ | □ |
| 29. Do you think you will seek help from all aspects to achieve your goals? | □ | □ | □ | □ | □ |
| 30. Do you think you will make a schedule to help yourself finish the task on time? | □ | □ | □ | □ | □ |
| 31. Do you think you will eventually achieve your goal even if it might take a long time? | □ | □ | □ | □ | □ |
| 32. Do you think you can achieve your goal? | □ | □ | □ | □ | □ |
| 33. Do you think whether you can become a leader mainly depends on your own abilities? | □ | □ | □ | □ | □ |
| 34. Do you think your future life depends on your own efforts? | □ | □ | □ | □ | □ |
| 35. Do you think you have the ability to acquire the knowledge you want or need? | □ | □ | □ | □ | □ |
| 36. Do you think you can finish any task well despite their difficulties? | □ | □ | □ | □ | □ |
| 37. Do you think IEE education is very important? | □ | □ | □ | □ | □ |
| 38. Do you think the teacher's teaching is interesting and challenging? | □ | □ | □ | □ | □ |
| 39. Do you think what the teacher teaches in class is of any use to you? | □ | □ | □ | □ | □ |

If you are willing to accept further interviews, please leave your contact information:

This is the end of the QS. Thank you for your cooperation! Wish you success in your work and study and happy life!
